# Malignant peripheral nerve sheath tumor of the nasal cavity and nasopharynx in a child: A case report

**DOI:** 10.1097/MD.0000000000014223

**Published:** 2019-01-25

**Authors:** Qian Li, Hongguang Pan, Lan Li, Juan Cao

**Affiliations:** a Zunyi Medical University, Zunyi, Guizhou; b Department of Otolaryngology, Shenzhen Children's Hospital, Shenzhen, Guangdong; c Department of Radiology, Shenzhen Children's Hospital, Shenzhen, Guangdong, China.

**Keywords:** malignant peripheral nerve sheath tumour, nasal cavity mass, nasopharynx, pediatric

## Abstract

**Rationale:**

Malignant peripheral nerve sheath tumors (MPNSTs) are rare neoplasms with only a few reported cases affecting the nasal cavity, paranasal sinuses, and anterior skull base.

**Patient concerns:**

A 12-year-old girl with a mass in her nose was admitted to the Department of Otorhinolaryngology of Shenzhen Children's Hospital. She had a 4-month history of progressive, unilateral right nasal obstruction, unilateral mucopurulent rhinorrhea, foul nasal odor, snoring, hyposmia, occasional epistaxis, and no headache, no facial numbness, without eye swelling and vision loss.

**Diagnose:**

A computed tomography (CT) scan of the paranasal sinuses showed a mass (right inflammatory polyp and calcification) involving the nasal cavity, the right maxillary sinusitis, ethmoid sinusitis. There was no nasal septum, orbital, or skull base involvement. On the basis of pathological and immunohistological findings, the tumor was diagnosed as an MPNST with heterogeneous components (cartilage and bone) mesenchymal differentiation after the operation.

**Interventions:**

The girl was treated by surgery and chemotherapy.

**Outcomes:**

The postoperative course was uneventful. There was no recurrence observed during the 3-year follow-up.

**Lessons:**

The primary MPNST in the nasal cavity is rare, if nasal neoplasms do not respond well to vasoconstrictors and glucocorticoids in children, the possibility of a tumor should be considered. If new organisms grow rapidly with hemorrhagic necrosis, the possibility of a malignant tumor is greater.

## 1 Introduction

Malignant peripheral nerve sheath tumor (MPNST) is defined as any malignant tumor arising from or differentiating toward the cells of the peripheral nerve sheath, except for tumors originating from the epineurium or the peripheral nerve vasculature.^[1,2]^ MPNSTs are among the most aggressive malignant tumors, have the highest local recurrence rate among sarcomas, and show a marked propensity for dissemination and metastasis. MPNSTs are rare neoplasms with only a few reported cases wherein the nasal cavity, paranasal sinuses, and anterior skull base were affected. The biological and clinical behaviors of this aggressive tumor are poorly understood.^[3–6]^ Nonspecific symptoms such as nasal congestion and rhinorrhea may persist for months to even years before a nasal mass is suspected. The differential diagnosis relative to the benign and malignant etiologies of a nasal mass in a child is broad, and a surgical biopsy is required for a definitive pathological diagnosis. Nasal polyps are often the first impression of nasal cavity mass in children. Here we present a case of MPNST in the nasal cavity and nasopharynx to further elucidate the natural history and prognosis of this rare neoplasm in the head and neck.

## 2 Case report

### 2.1 Clinical characteristics

**Figure 1:**
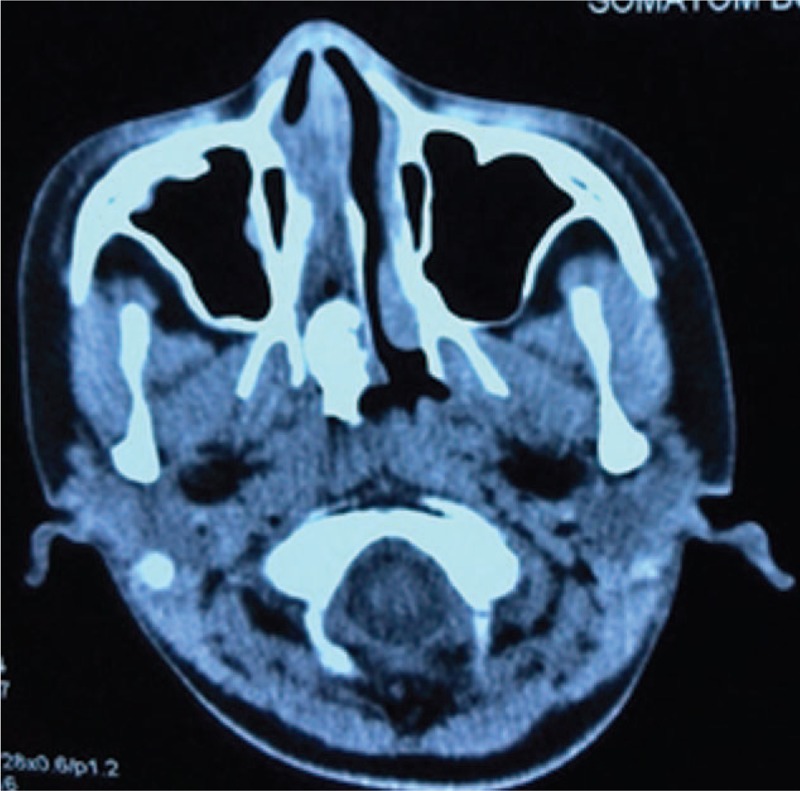
Axial CT of the paranasal sinuses showing the tumor mass affecting the right-sided nasal cavity and nasopharynx. A new bone-like material can be seen in the nasopharynx, without bony erosion involvement of the nasal septum, orbital, and skull base. CT = computed tomography.

A 12-year-old girl with a mass in her nose was admitted to the Department of Otorhinolaryngology of Shenzhen Children's Hospital in July 2015. She had a 4-month history of progressive, unilateral right nasal obstruction, unilateral mucopurulent rhinorrhea, foul nasal odor, snoring, hyposmia, and occasional epistaxis; there was no itching, sneezing, headache, facial numbness, eye swelling, vision loss, earache, or hearing loss. She first noted the presence of the painless mass in March 2015, and the mass gradually grew in size. A clinical examination revealed a painless mass in the right nasal cavity that was not sensitive to xylometazoline contraction. An anterior rhinoscopy showed a white polypoid neoplasm in the right nose. The anterior segment of the tumor was not smooth and filled the nasal cavity and nasopharynx. There was no swelling on the right side of the patient's face, no changes in the soft and hard palate, and eye movement was normal. The bilateral neck did not reach the enlarged lymph nodes. The patient's lungs had normal respiratory sounds. The liver and spleen were not enlarged or lumped. A computed tomography (CT) scan (Fig. 1) of the paranasal sinuses showed a mass (right inflammatory polyp and calcification) involving the nasal cavity, the right maxillary sinusitis, and ethmoid sinusitis. There was no nasal septum, orbital, or skull base involvement. A chest X-ray showed no abnormality in the lungs. A preoperative biopsy of the nasal cavity under topical anesthesia showed an inflammatory change. The initial diagnosis was a right nasal-nasopharyngeal space-occupying lesion. Hemorrhagic necrotizing polyps and ectopic teeth were suspected.

**Figure 2:**
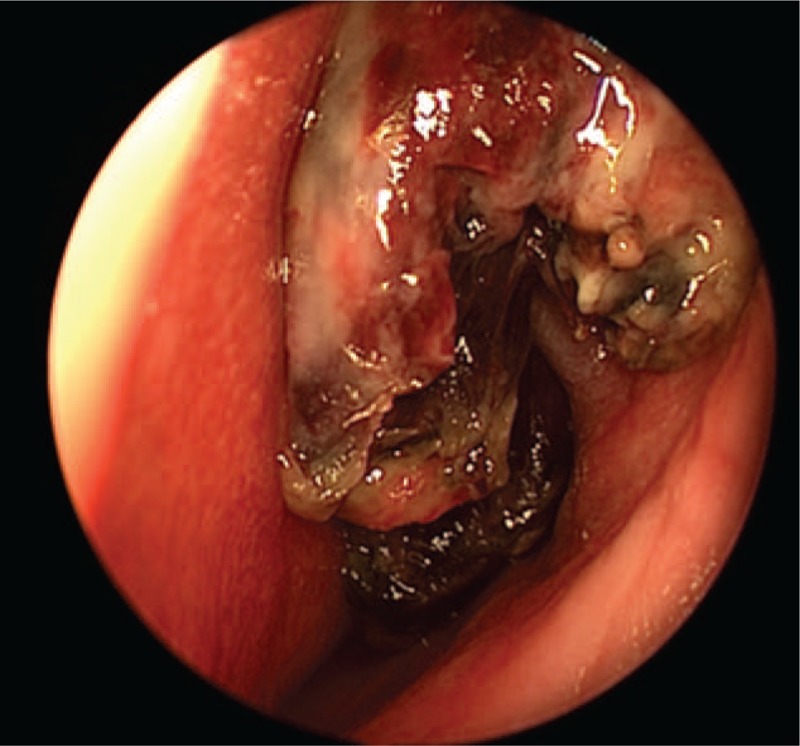
Erosive necrosis and defects (due to the preoperative biopsy) can be seen in the anterior segment of the tumor.

**Figure 3:**
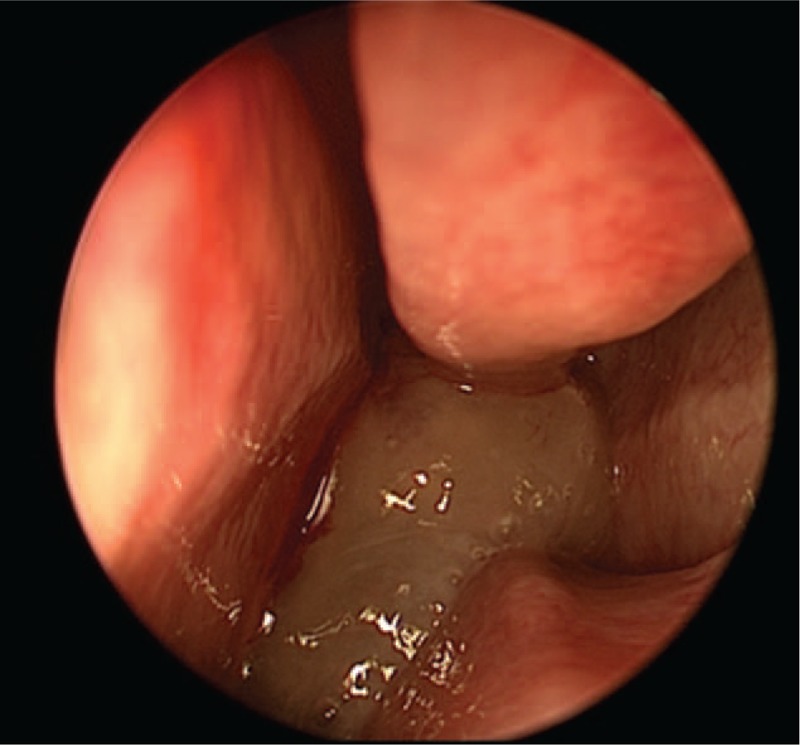
The nasopharyngeal neoplastic tissue was slightly yellowish and harder than a nasal polyp.

**Figure 4:**
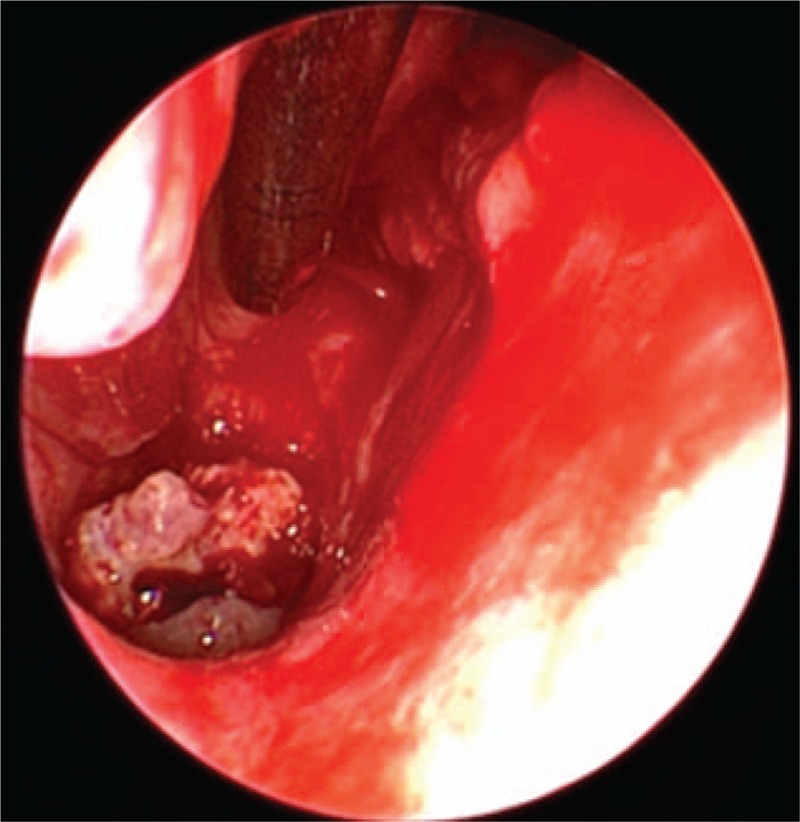
The nasopharyngeal mass was removed and the new bone at the bottom can be seen.

**Figure 5:**
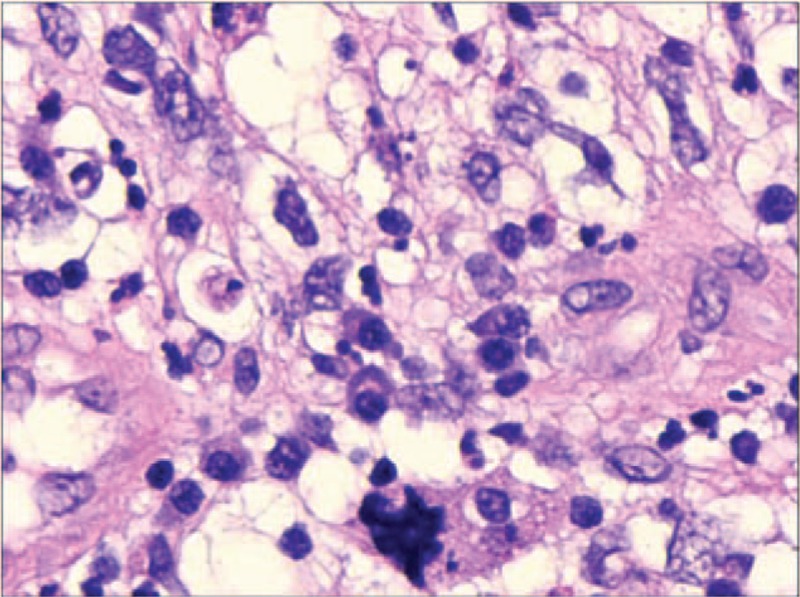
(HE 10^∗^40) Histological findings. The tumor exhibited hyper-cellularity and increased mitotic activity. A large number of spindle-shaped cells with a high nuclear-to-cytoplasmic ratio were arranged in short and long fascicles.

**Figure 6:**
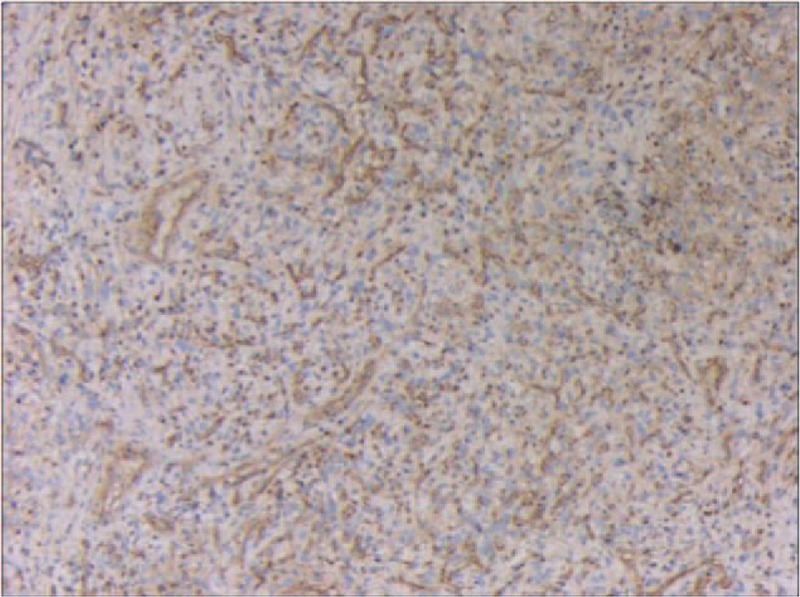
Immunohistochemical staining showing positive vimentin.

**Figure 7:**
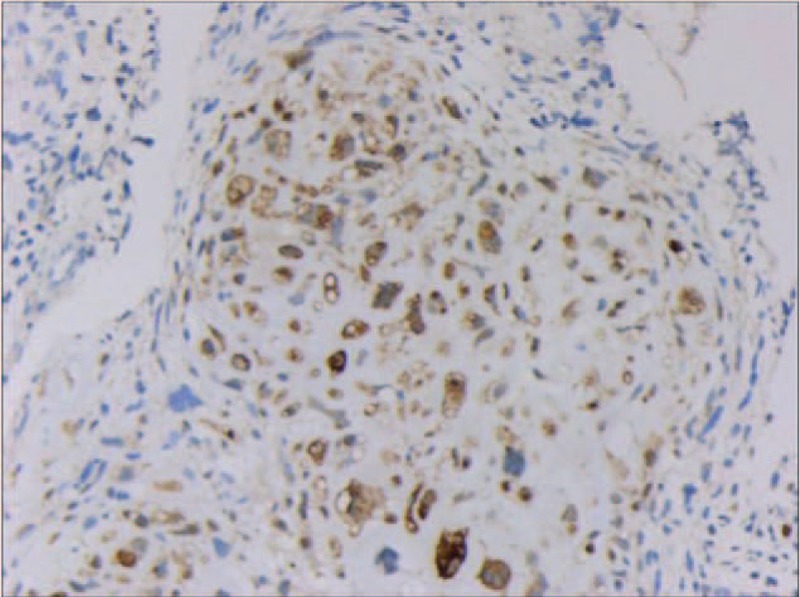
Immunohistochemical staining showing positive S-100. S-100 = soluble protein-100.

**Figure 8:**
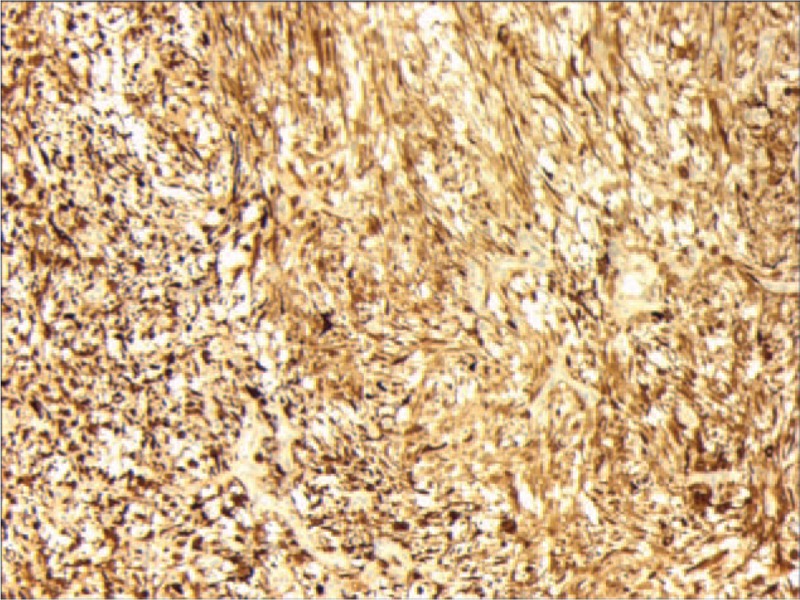
Immunohistochemical staining showing positive PGP9.5. PGP9.5 = protein gene product9.5.

A right nasal cavity-nasopharynx neoplasm resection was performed under general anesthesia on the fourth day after admission. During the operation, a polypoid tumor of the right nasal cavity was seen, including erosion and necrosis of the surface of the anterior segment of the tumor, completely blocking the right nasal cavity (Fig. 2). The tumor was removed with a microdebrider; there was less bleeding when the microdebrider was used to cut the nasal cavity and nasopharyngeal mass (Fig. 3). The tumor extended from the olfactory cleft to the entire nasal cavity and nasopharynx. No tumor was found after opening the anterior ethmoid sinus and maxillary sinus. Obstructive inflammation of the paranasal sinus was apparent. After resecting the neoplasms in the nasal cavity and nasopharynx, a grayish white bone (2 cm × 1.5 cm × 1 cm) was seen under the mass in the nasopharynx. The bone was loose (Fig. 4), and its surface was uneven; further, there was a small amount of bleeding in the bone bed wound. Microscopically, the tumor exhibited hypercellularity and increased mitotic activity. A large number of spindle-shaped cells with a high nuclear to cytoplasmic ratio were arranged in short and long fascicles (Fig. 5) with indistinct cytoplasmic border and varying degree of nuclear pleomorphism. Epithelial, cartilaginous, and bony heterologous elements were observed. The bone in the nasopharynx was an immature reticular trabecular bone-like material with increased and irregular adhesion lines and visible bone lacunae. Further immunohistochemical analysis revealed that the mesenchymal marker vimentin and the neuroectodermal marker soluble protein-100 (S-100) and protein gene product (PGP) 9.5 were positive (Figs. 6–8). The clinical diagnosis was nasal and nasopharyngeal sarcoma (T2N0M0). On the basis of these pathological and immunohistological findings, the tumor was diagnosed as an MPNST with heterogeneous components (cartilage and bone) mesenchymal differentiation. Pathological HE staining revealed spindle cells arranged in bundles or fascicles with obvious atypia and high mitotic rates; hence, a malignant tumor was suggested. Vimentin, S-100, and PGP 9.5 were positive.

**Figure 9:**
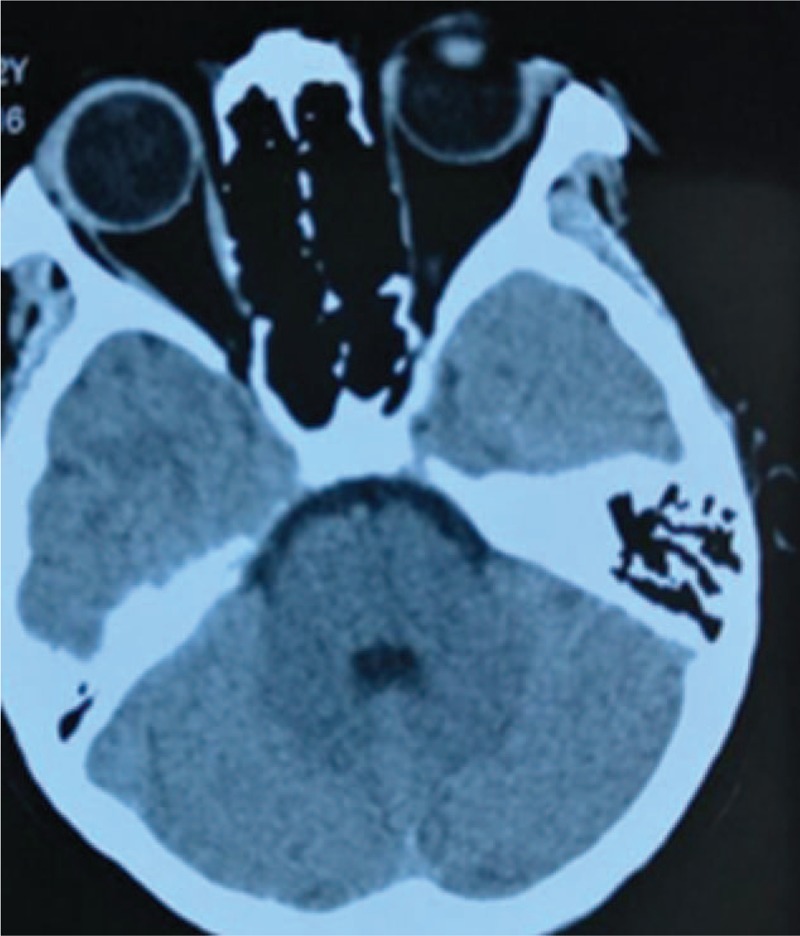
Axial CT of the paranasal sinuses showing no neoplastic recurrence in the right nasal cavity and nasopharynx at 9 months after surgery. CT = computed tomography.

Two weeks after the operation, the patient received intravenous chemotherapy at another hospital. Chemotherapy was administered using pirarubicin (THP), high-dose cisplatin (DDP), and high-dose methotrexate (HD-MTX). The patient received 8 courses of chemotherapy and was followed-up for more than 3 years postoperatively. To date, she remains healthy with no evidence of recurrence or metastasis. Her physical development is normal. She has good nasal ventilation and no runny nose, nosebleeds, headaches, facial numbness, impaired vision, cough, or asthma. An endoscopic examination showed no recurrence of nasal mass. The cavity was clean, there was no discharge in the nasal passage, and the nasopharyngeal mucosa was smooth. Recurrence of the tumor was not found on CT examinations performed at 9 months after the operation (Fig. 9). There was no sign of peripheral metastasis on PET/CT examination.

### 2.2 Informed consent

The patient's parents consented to the publication of this case report.

## 3 Discussion

Cancer of the nasal cavity in children is particularly challenging because of the often nonspecific symptoms and characteristic advanced degree of local destruction at presentation. Tumors of the nasal cavity are often initially misdiagnosed as nasosinusitis and treated empirically. MPNSTs are rare neoplasms with an estimated incidence of 0.1 per 100,000 per year in the general population.^[5]^ They are also known as neurofibrosarcomas, neurogenic sarcomas, malignant neurilemmomas, and malignant schwannomas. The term “malignant peripheral nerve sheath tumor” was adopted in the early 1990s by the World Health Organization Committee for the classification of soft tissue tumors. These tumors account for 5% to 10% of all soft tissue sarcomas, but comprise only 2% to 6% of head and neck sarcomas.^[7]^ These tumors occur at all ages. Valentin et al^[8]^ reported that the mean age of patients with tumors at diagnosis was 42 years old, The majority of tumors developed in the limbs, were deep-seated and of high grade. Reports of these tumors in children are rare, with no significant gender difference. Primary neurogenic tumors of the nose and paranasal sinuses are uncommon, accounting for approximately 4% of all neural tumors of the head and neck.^[9]^ The skull base is even less often affected. Involvement of the paranasal sinuses or the skull base in MPNST is extremely rare.^[6]^ The main manifestations of MPNST in the nasal-sinus are local occupying tumor, and early manifestations include nasal obstruction, pus discharge, nasal bleeding, and foul odor, and no distinctive manifestation. These tumors rarely extend to the intracranial and orbital regions, with no specific changes observed in the imaging analysis. The mass is insidious and easy to miss or misdiagnose, which is a challenge for pathologists and otolaryngologists.

The incidence of nasal polyps in children is low, and the occurrence of malignant tumors in the nasal cavity is low. As most ENT physicians have insufficient experience in treating malignant tumors in children, the condition is easy to misdiagnose and mistreat. Diagnosis is often delayed due to the presence of specific symptoms that simulate more common sinonasal conditions such as chronic sinusitis or polyps. It is well known that glucocorticoids have a certain therapeutic effect on nasal polyps and are generally used routinely before functional endoscopic sinus surgery (FESS) for nasal polyps. If nasal neoplasms do not respond well to vasoconstrictors and glucocorticoids, clinicians should consider the possibility of a tumor. If new lesions new organisms grow rapidly with hemorrhagic necrosis, the possibility of a malignant tumor is greater. Otorhinolaryngologists, especially pediatric otorhinolaryngologists, should pay close attention to nasal masses to avoid misdiagnosis and mistreatment. In our patient, a preoperative biopsy suggested inflammation (insufficient tissue may have been obtained under topical anesthesia), and the CT scan did not show lymph node enlargement in the neck and axilla or bone erosion hence, frozen pathology was not performed during the operation. Surgical resection was conducted in accordance with the principle of benign tumor removal without excessive removal of the surrounding bone. Surgical biopsy is precise for a histopathologic diagnosis, with a particular reliance on molecular studies and special staining for immunohistochemical markers. The cells of MPNST origin are not fully established. MPNSTs^[10]^ arising from the peripheral nerves are generally regarded as being Schwannian in origin. MPNSTs can histologically resemble other malignant tumors, particularly malignant melanoma and other spindle cell sarcomas. The increase in the S-100 protein level is a specific and sensitive biochemical marker of central nervous system damage; thus, S-100, PGP 9.5, and vimentin can be used as reliable pathological diagnostic indicators of MPNST. The diagnosis in our case was MPNST with heterogeneous components.

In the head and neck, a wide surgical excision implies en bloc resection of the involved soft tissue, muscle, and bone. The involved nerves should be followed proximally in an attempt to obtain clear margins.^[11]^ Because regional lymph node metastases are notably rare, prophylactic neck dissection is generally not recommended,^[12]^ and preventive cervical lymph node dissection is not necessary. The use of postoperative radiotherapy and chemotherapy in the treatment of MPNST is controversial, and most authors believe that the tumors are not sensitive to radiotherapy. Some authors posit that chemotherapy can reduce the recurrence rate of tumors and metastases to distant regions, improve the patients’ quality of life, but not prolong their overall survival time. Most physicians opt for radical resection and postoperative chemotherapy. In our present case, 2 weeks after the operation, the patient was treated with chemotherapy at another hospital. Chemotherapy was administered using pirarubicin (THP), high-dose cisplatin (DDP), and high-dose methotrexate (HD-MTX). Nine months later, after 8 courses of chemotherapy and endoscopy, a whole body PET/CT examination showed no tumor recurrence. This is inconsistent with reports in the literature that the local resection of nasal cavity MPNST is not completely curable. At present, 3 years after her operation, our patient remains healthy with no evidence of recurrence and metastasis.

## 4 Conclusions

MPNST is a rare disease occurring in the nasal-sinus sinuses and is even less common in children and adolescents. For nasal cavity with the clinical features of a malignant neoplasm, it is necessary to fully evaluate before operating, determine the surgical plan according to the frozen pathological results, confirm the diagnosis according to the pathological features after surgery, and provide comprehensive treatment. In pediatric cases, physicians should fully consider their patients’ physical development to preserve physiological functions and ensure long-term quality of life.

## Acknowledgments

The authors thank Professor Xiongzhen Zhu, Department of Pathology, Affiliated Tumor Hospital of Fudan University, for assistance with the pathological diagnosis.

## Author contributions

**Conceptualization:** Qian Li, Hongguang Pan, Juan Cao.

**Data curation:** Juan Cao, Qian Li.

**Methodology:** Hong Pan.

**Supervision:** Lan Li,Juan Cao.

**Validation:** Hongguang Pan.

**Visualization:** Hongguang Pan, Lan Li.

**Writing – original draft:** Qian Li, Hongguang Pan, Lan Li.

**Writing – review & editing:** Qian Li, Hongguang Pan, Lan Li.
